# Validity of Intraoperative Imprint Cytology and Frozen Section for Sentinel Lymph Node Assessment in Breast Cancer: A Single-Centre Experience

**DOI:** 10.7759/cureus.88784

**Published:** 2025-07-25

**Authors:** Kopinath Kathirvetpillai, Sharon R Constantine

**Affiliations:** 1 Histopathology, William Harvey Hospital, East Kent Hospitals University National Health Service Foundation Trust, Ashford, GBR; 2 Pathology, National Hospital of Sri Lanka, Colombo, LKA

**Keywords:** breast cancer, frozen section, metastasis, sentinel lymph node, touch imprint cytology

## Abstract

Background: Sentinel lymph node (SLN) evaluation is central to breast cancer management. Intraoperative touch imprint cytology (TIC) and frozen section (FS) are standard, rapid techniques; however, their performance compared to the gold standard of histopathology in Sri Lanka is underreported. This study evaluates the validity of intraoperative TIC and FS in identifying SLN metastases in patients with breast cancer.

Methods: We retrospectively analysed 416 SLN biopsies (2018-2020), with all cases assessed by TIC, FS, and subsequent paraffin histology. Demographic and clinicopathologic features were correlated with diagnostic metrics.

Results: Mean patient age was 55.9 years. Tumour sizes were ≤20 mm in 23.6%, 21-50 mm in 66.6%, and ≥50 mm in 9.9%. Most cases (92.1%) were invasive carcinoma of no special type (NST). Sensitivity and specificity for TIC were 90.6% and 100%; for FS, 92.7% and 100%. Combined TIC+FS yielded a sensitivity of 92.7% and a specificity of 100%. Detection rates for micrometastases were lower, and sensitivity for TIC was reduced in cases of lobular carcinoma and those where no LVI was found in the primary tumour.

Conclusion: Both TIC and FS individually showed high sensitivity (90.6% and 92.7%, respectively) and perfect specificity (100%) in the detection of metastatic deposits in SLNs. Combining TIC and FS maintained excellent specificity, while also maintaining the same sensitivity as FS alone. All three methods demonstrated consistently strong predictive values and overall accuracy, with limitations for smaller deposits and specific subtypes, such as invasive lobular carcinoma.

## Introduction

Breast cancer remains the most common malignancy among women worldwide and is a significant cause of cancer-related death in Sri Lanka [1,2]. The burden of breast cancer in Sri Lanka has been increasing, with over 3000 new cases identified in 2018 alone [1]. Advances in diagnostic strategies and multidisciplinary management have improved outcomes; however, accurate axillary staging remains critical for therapeutic decision-making [3,4].

Sentinel lymph node (SLN) assessment is the established standard for staging early breast cancer in patients with a clinically negative axilla [5,6]. Intraoperative SLN assessment allows immediate surgical decision-making, potentially sparing many patients a second procedure for axillary clearance, thus reducing morbidity [7,8]. Two widely used intraoperative techniques are touch imprint cytology (TIC) and frozen section (FS), both of which offer rapid results [9]. While FS provides tissue architecture and is highly specific, it is resource-intensive; in contrast, TIC is fast and cost-effective, but may have lower sensitivity for small metastases [10-13].

The combined use of TIC and FS is routine in many centres in Sri Lanka; however, local data on their diagnostic validity are sparse. This study aims to evaluate the sensitivity, specificity, and accuracy of TIC, FS, and their combination, while analysing clinicopathological factors affecting performance.

## Materials and methods

Study design and setting

A retrospective, descriptive cross-sectional study was conducted at the Department of Histopathology, National Hospital of Sri Lanka. Eligible cases with SLN biopsies reported between 2018 and 2020 were included to evaluate the diagnostic performance of intraoperative TIC and FS compared to final histopathology in the detection of SLN metastases in breast cancer patients.

Histopathological Methods

During gross examination, SLNs were sectioned at 2 mm intervals perpendicular to the long axis by a junior pathologist. TIC was performed on all exposed surfaces, with an average of six imprints per slice. FSs were prepared by a senior biomedical scientist, averaging three FSs per slice. All slides were stained with hematoxylin and eosin (H&E).

Study population

Inclusion criteria encompassed the cases of mastectomy and wide local excision with sentinel SLN biopsy, evaluated by both TIC and FS, with definitive diagnosis confirmed by paraffin histology. The cases where only TICs were performed, instances of damaged slides, and patients who had received neoadjuvant therapy were excluded. To our knowledge, there are no published studies from Sri Lanka reporting on the sensitivity and specificity of TIC and FS for SLN biopsy assessment in breast cancer. The sample size for this study was determined as 416 using the standard formula, based on an anticipated sensitivity of 85% and specificity of 95%.

Data collection

The extracted data included patient age, tumour size, Nottingham grade, tumour subtype, presence or absence of lymphovascular invasion (LVI) in the primary tumour, number and size of SLNs examined, final histology of SLN, and size of any metastatic deposit. TIC and FS slides were retrospectively systematically reviewed at all magnifications by the principal investigator and the supervisor, with particular attention paid to the subcapsular sinus. Both reviewers were blinded to the final histological diagnosis while assessing TIC and FS independently.

Statistical analysis

The data were analysed and presented as percentages and numbers by using descriptive statistics. Sensitivity, specificity, positive predictive value (PPV), negative predictive value (NPV), and accuracy were calculated for TIC, FS, and combined TIC+FS using final histology as the gold standard. Subgroup analyses evaluated performance by tumour size, histological subtype, and LVI status. Detection rates for macrometastases (>2 mm), micrometastases (0.2-2 mm), and isolated tumour cells (<0.2 mm) were calculated. Statistical analysis was performed using standard methods.

## Results

Demographic and tumour characteristics

The mean patient age was 55.9 years, with a range of 26-83 years. The mean tumour size was 29.8 mm with a range of 7-107 mm; the tumour sizes were ≤20 mm in 98 cases (23.6%), 21-50 mm in 277 cases (66.6%), and ≥50 mm in 41 cases (9.9%). Most cases were invasive carcinoma NST (no special type; 92.1%), followed by smaller proportions of lobular (3.1%) and micropapillary (2.9%) subtypes. Rare histological types were also observed at low frequencies. A summary is shown in Table 1.

**Table 1:** Histological Subtypes NST, no special type.

Subtype	Cases (n)	Percentage
Invasive carcinoma NST	383	92.1
Invasive lobular carcinoma	13	3.1
Invasive micropapillary carcinoma	12	2.9
Mucinous carcinoma	4	1.0
Invasive cribriform carcinoma	2	0.5
Invasive papillary carcinoma	1	0.2
Invasive tubular carcinoma	1	0.2

Among the cases, grade 2 tumours (NG2) were the most common (44.5%) and showed the highest nodal metastasis rate (25.9%). Grade 1 (NG1) and grade 3 (NG3) tumours had metastasis rates of 19.4% and 21.3%, respectively. These findings are summarised in Table 2.

**Table 2:** Nottingham Grade and Nodal Status NG, Nottingham Grade.

Grade	Cases (n)	Percentage	With Metastasis (n)	Metastatic Rate (%)
NG1	62	14.9	12	19.4
NG2	185	44.5	48	25.9
NG3	169	40.6	36	21.3

LVI was observed in 13.5% of cases and was associated with a larger mean tumour size (35.5 mm) compared to those without LVI (28.9 mm), indicating its correlation with more aggressive disease. SLNs were examined per case, ranging from one to four nodes. Most cases involved one (238 cases) or two nodes (117 cases), with fewer cases having three or four nodes. The average SLN size was 10.4 mm, with a range of 3 to 35 mm. The mean size of metastatic deposits among positive nodes was 4.59 mm, ranging from 0.4 mm to 11.0 mm.

Diagnostic performance

The accuracy of TIC, FS, and their combined application was evaluated using final histopathology as the gold standard. Both TIC and FS individually showed high sensitivity (90.6% and 92.7%, respectively) and perfect specificity (100%). Both techniques showed substantial concordance with the final histopathological results (κ = 0.93 for TIC and κ = 0.95 for FS). Combining TIC and FS maintained excellent specificity, while also maintaining the same sensitivity as FS alone. All three methods demonstrated consistently strong predictive values and overall accuracy, which emphasises their effectiveness for real-time evaluation of SLNs during surgery. Table 3 presents a comparison of sensitivity, specificity, PPV, NPV, and diagnostic accuracy for TIC, FS, and the combined method.

**Table 3:** Diagnostic Performance of TIC, FS, and Combined TIC+FS PPV, positive predictive value; NPV, negative predictive value; TIC, touch imprint cytology; FS, frozen section.

Method	Sensitivity (%)	Specificity (%)	PPV (%)	NPV (%)	Accuracy (%)
Touch imprint cytology	90.6	100	100	97.3	97.8
Frozen section	92.7	100	100	97.9	98.3
Combined TIC + FS	92.7	100	100	97.9	98.3

Factors affecting diagnostic accuracy

Metastasis Size

The detection rates of metastatic deposits by TIC and FS were also analysed according to the size of the metastases. These are stratified into macrometastases, micrometastases, and isolated tumour cells. Table 4 presents the comparative performance.

**Table 4:** Diagnostic Accuracy by Metastasis Size TIC, touch imprint cytology; FS, frozen section.

Metastasis Size	TIC Detection Rate (%)	FS Detection Rate (%)
Macrometastases (>2 mm)	98.8	100
Micrometastases (0.2-2 mm)	36.4	45.5
Isolated tumour cells (<0.2 mm)	0	0

Tumour Subtype

Sensitivity for TIC was slightly lower in invasive lobular carcinoma (85.7%) compared to NST (90.7%).

Lymphovascular Invasion (LVI)

Sensitivity for TIC was 100% when LVI was present and 79.5% when LVI was absent.

## Discussion

This large, single-centre study confirms that both TIC and FS, individually and in combination, offer high specificity (100%) and robust sensitivity for intraoperative detection of SLN metastasis in breast cancer. These findings are comparable to, or slightly exceed, most published international series [14,15]. For example, Wang et al. [15] reported a TIC sensitivity of 76.2% and Petropoulou et al. [14] found an FS sensitivity of 80%.

Both methods were highly reliable for macrometastases but showed lower detection rates for micrometastases (TIC 36.4%, FS 45.5%). Neither method reliably detected isolated tumour cells, mirroring the limitations noted in previous studies [16,17]. The combination of TIC and FS maintained sensitivity, similar to FS alone, suggesting that FS may be prioritised in well-resourced centres, with TIC remaining a valuable adjunct, particularly where FS is unavailable or where technical challenges limit FS quality [18-26]. 

Subgroup analyses revealed that TIC is somewhat less sensitive for invasive lobular carcinoma compared to NST (85.7% vs. 90.7%), consistent with international reports [27,28]. The lower detection in lobular carcinoma likely reflects its subtle cytological features and discohesive growth pattern, which complicate intraoperative cytological diagnosis [28]. Sensitivity was also higher (100%) in cases with LVI, likely due to the presence of larger and more easily identifiable nodal deposits.

Our results support the ongoing use of intraoperative SLN assessment in Sri Lanka, particularly given the high accuracy for clinically significant (macrometastatic) disease. Nevertheless, as with global practice, some micrometastatic and isolated tumour cell disease will be missed intraoperatively, underlining the importance of postoperative histological assessment and multidisciplinary discussion [5,6,9].

Strengths and limitations

This study represents the largest local dataset examining the SLNs assessed intraoperatively for breast cancer, and benefits from review by experienced histopathologists. All procedures were carried out under well-defined protocols, ensuring consistency and reliability in specimen handling and diagnostic evaluation. This comprehensive approach provides valuable data, as there is a paucity of research on this subject in Sri Lanka.

However, several limitations must be acknowledged. The retrospective design may produce inherent biases, as data collection and case selection were dependent on existing records rather than prospective recruitment. The exclusion of cases in which patients who had received neoadjuvant therapy may limit the applicability of the results to broader patient populations. Furthermore, routine immunohistochemical staining was not performed on all nodal specimens, potentially leading to underdetection of isolated tumour cells. Although methods were developed to minimise interobserver variability through double reporting, the possibility of subjective interpretation, particularly the experience of a pathologist, remains and could influence diagnostic outcomes. Despite these challenges, the study offers valuable insights and identifies areas for future improvement in local practice.

Comparison with literature

Our findings are closely aligned with previously published data from regional and international studies, which have reported TIC sensitivity ranging from 69% to 92% and FS sensitivity ranging from 70% to 88%, with high specificity in all series [14-26]. The challenge of detecting micrometastases and isolated tumour cells intraoperatively is universal [20-26].

## Conclusions

This study highlights the importance of both TIC and FS in assessing SLNs during breast cancer surgery. While FS remains the most accurate method, TIC stands out as a cost-effective and practical alternative, particularly in resource-limited environments. 

The combined use of TIC and FS is most advantageous in settings where both are available, ensuring a thorough evaluation of SLNs that supports more confident intraoperative decisions and reduces the need for additional surgeries. However, TIC alone can still be a reliable and affordable choice in institutions with fewer resources, despite some challenges in identifying micrometastases and isolated tumour cells. These challenges are particularly evident in specific cancer subtypes, such as invasive lobular carcinoma.

In summary, both TIC and FS contribute meaningfully to the detection of metastatic disease, especially larger metastatic deposits, and their thoughtful application can improve patient care by guiding surgical decisions more effectively. Continued refinement of these approaches is encouraged to address current limitations and further advance the accuracy of intraoperative diagnosis in breast cancer.
